# High Density of Tree-Cavities and Snags in Tropical Dry Forest of Western Mexico Raises Questions for a Latitudinal Gradient

**DOI:** 10.1371/journal.pone.0116745

**Published:** 2015-01-23

**Authors:** Leopoldo Vázquez, Katherine Renton

**Affiliations:** 1 Posgrado en Ciencia Biológicas, Instituto de Biología, Universidad Nacional Autónoma de México, Mexico City, Distrito Federal, Mexico; 2 Estación de Biología Chamela, Instituto de Biología, Universidad Nacional Autónoma de México, San Patricio-Melaque, Jalisco, Mexico; DOE Pacific Northwest National Laboratory, Joint Global Change Research Institute, United States.

## Abstract

It has been suggested that a latitudinal gradient exists of a low density of snags and high density of naturally-formed tree-cavities in tropical vs. temperate forests, though few cavities may have characteristics suitable for nesting by birds. We determined snag and cavity density, characteristics, and suitability for birds in a tropical dry forest biome of western Mexico, and evaluated whether our data fits the trend of snag and cavity density typically found in tropical moist and wet forests. We established five 0.25-ha transects to survey and measure tree-cavities and snags in each of three vegetation types of deciduous, semi-deciduous, and mono-dominant *Piranhea mexicana* forest, comprising a total of 3.75 ha. We found a high density of 77 cavities/ha, with 37 cavities suitable for birds/ha, where density, and characteristics of cavities varied significantly among vegetation types. Lowest abundance of cavities occurred in deciduous forest, and these were in smaller trees, at a lower height, and with a narrower entrance diameter. Only 8.6% of cavities were excavated by woodpeckers, and only 11% of cavities were occupied, mainly by arthropods, though 52% of all cavities were unsuitable for birds. We also found a high density of 56 snags/ha, with greatest density in deciduous forest (70 snags/ha), though these were of significantly smaller diameter, and snags of larger diameter were more likely to contain cavities. The Chamela-Cuixmala tropical dry forest had the highest density of snags recorded for any tropical or temperate forest, and while snag density was significantly correlated with mean snag dbh, neither latitude nor mean dbh predicted snag density in ten forest sites. The high spatial aggregation of snag and cavity resources in tropical dry forest may limit their availability, particularly for large-bodied cavity adopters, and highlights the importance of habitat heterogeneity in providing resources for primary and secondary cavity-nesters.

## Introduction

Tree-cavity availability is frequently considered a limiting factor for reproduction of secondary cavity-nesting birds [1], which use existing cavities for nesting. More specifically, birds may be limited by the availability of cavities with characteristics suitable for nesting [2, 3]. In northern temperate forests, woodpeckers are important primary cavity-nesters, excavating 77% of cavities used by a variety of secondary cavity-nesters [4, 5, 6, 7]. In contrast, natural processes of damage and decay may be the main cause of cavity formation in tropical forests [7, 8].

The abundance of standing dead trees, or snags, may also be of importance for cavity-nesting birds, as these tend to be preferred substrates for primary excavators such as woodpeckers [3]. Gibbs et al. [9] found a higher density of snags in temperate forests compared to tropical and subtropical forests, and a greater proportion of cavity-nesting bird species in temperate forests are primary excavators. By comparison, density of tree-cavities is higher in forests closer to the equator [10], and these are predominantly naturally-formed, possibly as a result of warm, humid conditions accelerating decay processes [7]. Tropical forests maintain a greater diversity of cavity-nesting bird species, many of which are secondary cavity-nesters [9], and also maintain large secondary cavity-nesting species such as toucans, falcons, parrots, and macaws, all of which require large nest-cavities that tend to be naturally-formed [11, 8].

There is a scarcity of information on snag and tree-cavity resource availability in tropical forest, with only a few studies conducted at scattered sites, the majority in moist or wet forests [9, 10]. A comparison of temperate wet and dry forests in Tasmania found that wet forests had more cavities than dry forests, and that this may be related to the size, age and senescence of trees [12]. The tropical and subtropical dry broadleaf forest biome [13], known as tropical dry forest, experiences high seasonality in rainfall with an extended dry season [14], and is distributed mainly in the Americas [15]. Almost all of the remaining areas of tropical dry forest are now threatened by pressures from human activities of climate change, fragmentation, agricultural conversion, and human population growth [15]. Among these, fragmentation and climate change are the main factors affecting most areas of tropical dry forest in the Americas [15]. This is of concern as tropical dry forests maintain high species richness and are centers of endemism [16]. In particular, tropical dry forests maintain avifauna with a high percent of cavity-nesting bird species, many of which are threatened [17].

In the present study we determined the availability and characteristics of tree cavities and standing dead trees in a tropical dry forest of western Mexico. We evaluated whether woodpecker excavation or natural decay processes were the main origin of cavities in tropical dry forest. In particular, we aimed to determine whether tropical dry forest follows the latitudinal relationships of lower snag and higher cavity densities compared to temperate forests [9, 10]. If tropical dry forest follows the patterns exhibited by tropical moist and wet forests, then we would expect a density of about 20 snags/ha [9], and 50–60 tree-cavities/ha [10], though we also expected that a limited number of cavities would have characteristics suitable for nesting by birds [2, 3].

## Methods

### Study site

We conducted the study in the tropical dry forest biome [13] of the 132-km^2^ Chamela-Cuixmala Biosphere Reserve (19°22’ to 19°35’N; 104°56’ to 105°03’W), on the coast of Jalisco, Mexico. The reserve conserves an area of intact tropical dry forest which still maintains original vegetation cover [18, 19]. Mean annual precipitation is 748 mm, 85% of which falls in June to October, with a 6–8 month dry season [20]. The site has a hilly topography from 20 m to 420 m asl, where the dominant vegetation is dry deciduous forest on the hills and slopes, with patches of semi-deciduous forest in larger valleys [21]. Discontinuous patches of mono-dominant forest of *Piranhea mexicana* also occur at low elevations within 10 km along the Pacific coast of Nayarit to Michoacan [22].

Deciduous forest is characterized by a canopy height of 8–12 m and dense undergrowth, where the majority of plants drop their leaves for 5–8 months of the year [23]. Species composition of deciduous forest is diverse, with common tree species of *Amphipterygium adstringens, Bursera instabilis, Caesalpinia eriostachys, Ceiba aesculifolia, Cordia alliodora, Cordia eleagnoides, Croton pseudoniveus, Jatropha* spp., *Lonchocarpus* spp., *Lysiloma microphyllum*, and *Trichilia trifolia* [21, 24]. By comparison, semi-deciduous forest has a canopy height of 15–30 m, with trees having thick, straight trunks, where most trees retain their leaves, or drop leaves for only 1–3 months of the year [23]. Species composition of semi-deciduous forest is distinct to that of deciduous forest, and common tree species of semi-deciduous forest are *Astronium graveolens, Brosimum alicastrum, Bursera arborea, Couepia polyandra, Cynometra oaxacana, Ficus insipida, Sideroxylon capiri, Tabebuia donnell-smithii, Tabebuia rosea*, and *Thouinidium decandrum* [21, 24]. In contrast with the high diversity of deciduous and semi-deciduous forest types, the mono-dominant forest type is dominated by *Piranhea mexicana* at most size classes of trunk diameter [22].

### Placement of cavity survey transects

**Figure 1:**
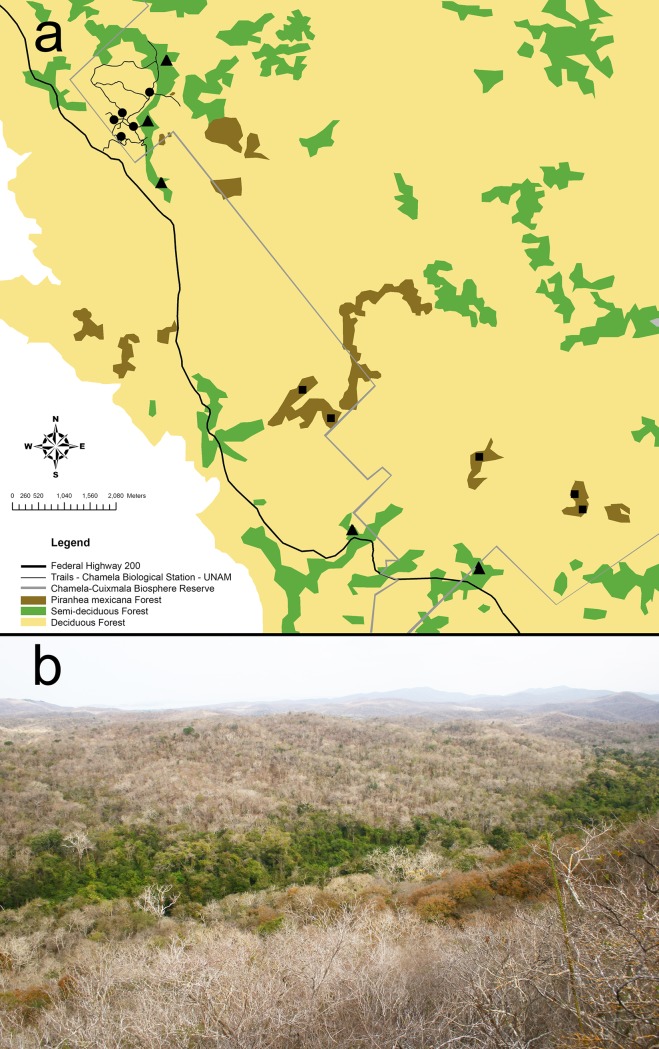
Map of the study site. This shows a) location of cavity transects in three vegetation types of deciduous forest (circles), semi-deciduous forest (triangles), and mono-dominant *Piranhea mexicana* forest (squares); and b) image taken in the dry season illustrating heterogeneity of vegetation types in tropical dry forest with deciduous forest on the hills, green semi-deciduous forest along the valley, and in the foreground a small patch of *Piranhea mexicana* forest with brown leaves (Photograph: Leopoldo Vázquez).

Research permits for the study were provided by the Secretaría del Medio Ambiente y Recursos Naturales, Mexico, and field studies did not involve endangered or protected species. We conducted surveys to determine the availability of tree-cavities and standing dead trees (snags) during May to November of 2009 in three vegetation types (Fig. 1): 1) deciduous forest, 2) semi-deciduous forest, and 3) mono-dominant *Piranhea mexicana* forest [21, 22]. We surveyed deciduous forest during the rainy season of August-November to ensure that trees had leaves for species identification and to avoid erroneous classification of standing dead trees. All survey transects were placed in undisturbed forest of the Chamela-Cuixmala Biosphere Reserve for each of the three vegetation types (Fig. 1A).

For the location of survey transects in different vegetation types, we identified large patches of semi-deciduous forest from Google Earth satellite images taken 30 April 2009 during the dry season, where semi-deciduous forest has a darker color as most trees retain leaf-cover in the dry season (Fig. 1B). We confirmed the presence of semi-deciduous forest identified from this initial inspection by visiting sites on the ground, whereby transects to survey semi-deciduous forest were placed along the streams known as Colorado, Zarco, Teopa, and Limbo. Mono-dominant *Piranhea mexicana* forest is indistinguishable from semi-deciduous forest in satellite images, as *Piranhea mexicana* trees also retain leaf-cover during the dry season, and occur mainly along valleys. Therefore, these vegetation patches were identified from a schematic diagram provided by Martijena and Bullock [22], combined with knowledge of the study site and inspection in the field. The largest areas of mono-dominant *Piranhea mexicana* forest occur along the Careyes stream and watersheds leading to the Cuixmala River, therefore transects for this vegetation type were located in these areas. Deciduous forest is the predominant vegetation on the hills but is difficult to access in most areas, therefore the trail system around the Chamela Biological Station was used to access areas of deciduous forest for surveys.

In each vegetation type we established five transects of 100 × 25 m [9], covering an area of 1.25 ha per vegetation type, with a total of 3.75 ha [25]. For surveys in semi-deciduous and monospecific *Piranhea mexicana* forest, which are restricted to small patches along stream valleys, we gained access to the specific vegetation patches by walking along dry stream-beds, and placed transects within the forest, off-set from the stream but in a direction parallel to the stream-bed, so as to avoid moving into deciduous forest on surrounding hills and slopes. For surveys in deciduous forest on the hills, we obtained access via forest trails, and placed transects within the forest offset from the trail. We delimited transect survey areas with pre-measured lengths of cord, marked at 10-m intervals with colored flagging, and used compass bearings to mark-out the survey area.

### Cavity availability and characteristics

Two people worked within the marked-out transect survey area to register all standing dead trees, and surveyed all trees with binoculars to detect cavity entrances. On locating a suspected cavity entrance, this was inspected and measured, as outlined below, to ensure that the entrance actually opened-out into a cavity, and complied with minimum depth criteria. We defined a cavity as a hole in a tree with an entrance opening ≥2 cm wide, and an internal cavity depth ≥8 cm [25]. Under this criteria, entrances which led to an opening of <8 cm deep were not recorded as cavities in the surveys. Furthermore, only tree openings with closed walls and a base were classified as cavities, hence open scars were not considered cavities, unless they had an enclosed depth of ≥8 cm from the bottom lip of the opening to the cavity floor. In the case of cavities with more than one entrance, we recorded the width of the largest entrance as this would determine the potential occupants or predators that could enter the cavity.

Cavities were categorized by tree condition (living or dead) and by origin (excavated or natural). Cavities excavated by woodpeckers were distinguished by the round symmetrical form of the cavity entrance, whereas natural cavities present an irregular entrance and may be localized in damaged areas of the tree, where a branch may have broken-off [6]. We recorded the species and diameter at breast height (dbh) of the tree supporting the cavity. Where a tree contained more than one cavity we recorded the dbh only once. We registered the location (branch or trunk) of the cavity entrance, and visually estimated the vertical inclination (0–90°) of the structure (trunk or branch) supporting the cavity [26]. In this way, a cavity on the straight vertical trunk of a tree had a vertical inclination close to 0, whereas a cavity on a horizontal branch would have an inclination closer to 90°. For horizontally orientated cavities we used a retractable tape-measure to determine horizontal depth.

Where possible we measured the characteristics of all cavities initially located from the ground. For cavities <4 m from the ground we used a ladder to measure cavity characteristics of height from the ground, support diameter of the trunk or branch where the cavity was located, entrance width, and cavity depth. For cavities above 4 m we used a digital dendrometer Criterion RD 1000 to measure entrance width, and support diameter, as well as to determine the height of cavities >15 m above the ground. In the case of cavities between 4 to 15 m above the ground, we used a tree-measuring pole with extension to 15 m, to measure cavity height. In order to measure cavity depth, we attached a fishing reel to the base of the measuring pole, with the nylon line extending up the measuring pole, through a horizontal PVC tube affixed to the top of the pole, to a lead weight at the end of the fishing line. The horizontal tube could then be inserted through the cavity entrance, and the lead weight lowered by feeding the line from the fishing reel. The distance by which the lead weight descended could be measured along the fishing line to determine the internal depth of the cavity. For logistical and safety reasons, it was not possible to measure the depth of cavities greater than 15 m above the ground. To determine the abundance of cavities suitable for birds, we considered only those cavities ≥2.5 m height and ≥13 cm depth [2], with a vertical inclination within the range of 0 to 45 degrees. We also registered the occupants of cavities whenever these were encountered.

Finally, we recorded all standing dead trees or snags, irrespective of size, within each survey transect, measuring the dbh of standing dead trees and their total height. Where a dead tree branched near the base we used the mean dbh and height of all dead trunks as the measurement for that tree. To enable comparison with data provided by Gibbs et al. [9] on snag density at ten forest sites, we considered in the analysis only snags with ≥10 cm dbh and ≥2 m height [9].

### Statistical analyses

We applied the Kolmogorov-Smirnov test of normality to determine whether data complied with a normal distribution for parametric tests, and applied non-parametric tests where the data deviated from a normal distribution. Data conformed to a normal distribution for total number per transect of all available cavities (K-S_15_ = 0.114, p = 0.20), cavities suitable for birds (K-S_15_ = 0.18, p = 0.20), all dead trees (K-S_15_ = 0.215, p = 0.06), and snags ≥10 cm dbh, ≥2 m height (K-S_15_ = 0.13, p = 0.20). Therefore, we applied one-way ANOVA with Tukey post-hoc comparison to determine differences in abundance of cavities and standing dead trees among vegetation types.

Cavity characteristics did not present a normal distribution for tree dbh (all cavities: K-S_223_ = 0.102, p < 0.001; bird cavities: K-S_122_ = 0.096, p = 0.008), support diameter (K-S_289_ = 0.098, p < 0.001; bird cavities: K-S_154_ = 0.097, p = 0.001), cavity height (all cavities: K-S_289_ = 0.132, p < 0.001; bird cavities: K-S_154_ = 0.138, p < 0.001), entrance width (all cavities: K-S_289_ = 0.115, p < 0.001; bird cavities: K-S_154_ = 0.106, p < 0.001), or cavity depth (all cavities: K-S_246_ = 0.274, p < 0.001; bird cavities: K-S_120_ = 0.281, p < 0.001). Characteristics of standing dead trees also did not conform to a normal distribution for tree dbh (all dead stumps: K-S_418_ = 0.159, p < 0.001; snags: K-S_211_ = 0.157, p < 0.001) or total height (all dead stumps: K-S_418_ = 0.181, p < 0.001; snags: K-S_210_ = 0.167, p < 0.001). Therefore, we used Kruskal-Wallis ANOVA with Dunn post-hoc analysis to compare characteristics of cavities and standing dead trees among vegetation types [27]. We also applied chi-square contingency table to evaluate whether cavity origin (natural or excavated) and suitability of cavities for use by birds was associated with vegetation type.

We used multiple logistic regression to determine the probability that a standing dead tree would contain a cavity (cavity = 1, no cavity = 0) based on dbh and height. We calculated the Wald statistic to determine which variable best predicted cavity presence [28]. Finally, we combined our data on snag density and mean dbh at the Chamela-Cuixmala site with that presented by Gibbs et al. [9] for ten temperate and tropical forest sites, as well as snag density at one tropical site provided by Boyle et al. [10], and applied Pearson’s correlation analyses to evaluate the proposed latitudinal relationship for snag density (n = 12 sites), and mean dbh (n = 10 sites with data on snag dbh). We also applied a multiple regression model to determine whether latitude or mean dbh were reliable predictors of snag density at the ten forest sites that provided both density and dbh data ([9], this study). Descriptive statistics are presented as mean with standard deviation, and we used the alpha < 0.05 significance level for all statistical analysis, which were conducted using R version 3.1.1., with the aod package [29, 30].

## Results

### Cavity availability and characteristics

We located a total of 289 cavities in 3.75 ha of tropical dry forest, though cavity abundance varied significantly among the three vegetation types (Table 1). The greatest density of 99 cavities/ha occurred in mono-dominant forests of *Piranhea mexicana*, which was similar to the 88 cavities/ha in semi-deciduous forest, but differed significantly from the density of 44 cavities/ha in deciduous forest (q = 13.8, p = 0.023).

**Table 1:** Habitat variation in tree-cavity and snag density.

	**Semi-deciduous forest**	***Piranhea* forest**	**Deciduous forest**	**Significance test**
**All cavities**	22 ± 6.4	24.8 ± 8.3	11 ± 6.3*	F_2,12_ = 5.4, p = 0.022
**Cavities suitable for birds**	10 ± 4.2	16.2 ± 4.3	4.6 ± 2.4***	F_2,12_ = 12.8, p = 0.001
**All dead stumps**	11.8 ± 5.2	20.0 ± 8.0	51.8 ± 14.1***	F_2,12_ = 23, p < 0.001
**Snags** (≥10 cm dbh, ≥2m height)	10.2 ± 5.2	14.4 ± 6.6	17.6 ± 4.4	F_2,12_ = 2.3, p = 0.14

Tukey post-hoc comparison: *p < 0.05; **p < 0.01; ***p < 0.001.

Mean (±SD) number of cavities and standing dead trees per transect in three vegetation types of tropical dry forest, with one-way ANOVA significance values.

Trees with cavities had a mean 31 ± 18 cm (range 5–110 cm, n = 223) dbh, and 21.5 ± 10.5 cm (range 5–65 cm, n = 289) support diameter at the cavity entrance. Cavities occurred at a mean height of 5.9 ± 4.9 m (range 0.2–26 m, n = 289) from the ground, with entrance diameter 8.2 ± 4.9 cm (range 2–31.4 cm, n = 289), and vertical cavity depth 49.0 ± 68.2 cm (range 8–660 cm, n = 246). However, cavity characteristics varied significantly among vegetation types (Table 2). In particular, cavities in deciduous forest occurred in trees of a smaller diameter, at a lower height from the ground, and with a narrower entrance diameter (Table 2).

**Table 2:** Habitat variation in characteristics of tree-cavities and snags in tropical dry forest.

	**Semi-deciduous forest**	***Piranhea* forest**	**Deciduous forest**	**Significance test**
**All cavities**				
DBH (cm)	37.5 ± 21.1	32.4 ± 15.5	18.7 ± 9.4***	H_2,223_ = 41.5, p < 0.001
Support diameter (cm)	23.0 ± 10.5	22.6 ± 10.9	16.1 ± 7.5***	H_2,289_ = 22, p < 0.001
Cavity height (m)	6.8 ± 5.9	6.6 ± 4.3	2.4 ± 1.4***	H_2,289_ = 46, p < 0.001
Entrance width (cm)	9.6 ± 5.7	8.4 ± 4.2	5.0 ± 3.2***	H_2,289_ = 44, p < 0.001
Internal depth (cm)	38.2 ± 54.5*	54.5 ± 77.8	55.0 ± 65.5	H_2,246_ = 7.7, p = 0.022
**Cavities suitable for birds**				
DBH (cm)	44.8 ± 23.8	36.9 ± 15.3	21.5 ± 9.8***	H_2,122_ = 25, p < 0.001
Support diameter (cm)	23.3 ± 9.4	23.9 ± 9.7	15.7 ± 5.3***	H_2,154_ = 15, p < 0.001
Cavity height (m)	9.9 ± 6.3	8.2 ± 4.1	3.6 ± 1.2***	H_2,154_ = 36, p < 0.001
Entrance width (cm)	10.8 ± 6.0	9.2 ± 3.7	5.0 ± 2.3***	H_2,154_ = 28, p < 0.001
Internal depth (cm)	40.6 ± 50.2	63.7 ± 88.4	68.0 ± 75.3	H_2,120_ = 2.7, p = 0.25
**All dead stumps**				
DBH (cm)	21.7 ± 9.4	16.5 ± 9.0	9.5 ± 5.1***	H_2,419_ = 133, p < 0.001
Total height (m)	5.8 ± 3.5	5.1 ± 2.7	4.4 ± 1.7***	H_2,418_ = 5.7, p = 0.059
**Snags** (≥10cm dbh,≥2m height)				
DBH (cm)	22.9 ± 9.3	19.7 ± 8.6	14.6 ± 5.4***	H_2,211_ = 43, p < 0.001
Total height (m)	6.4 ± 3.4	5.7 ± 2.8	5.1 ± 2.0	H_2,211_ = 3.5, p = 0.17

Dunn post-hoc comparison: *p < 0.05; **p < 0.01; ***p < 0.001.

Bonferroni adjusted significance level: cavities p = 0.005; snags p = 0.0125

Mean (±SD) characteristics of cavities and standing dead trees in three vegetation types of tropical dry forest, with Kruskal-Wallis significance values.

The vast majority of the cavities were formed naturally, with only 25 cavities (8.6% of all cavities) excavated by woodpeckers. However, cavity origin was significantly associated with vegetation type (χ^2^_2_ = 6.8, p < 0.05). Cavities excavated by woodpeckers were concentrated in semi-deciduous (11.8% of cavities) and *Piranhea* forest (9.7% of cavities), whereas all cavities registered in deciduous forest were formed naturally.

The majority of cavities occurred in live trees (80%), with only 54 cavities (19.7%) in standing dead trees. We recorded cavities in 32 tree species, though the most frequent tree species was *Piranhea mexicana*, with 76 cavities (26.3%), while the tree species of *Cyanometra oaxacana, Caesalpinia sclerocarpa*, and *Tabebuia* spp. all presented 5–6% of cavities each. Cavities were evenly distributed on branches (54% of cavities) or trunks (47%) of trees. The majority of cavities had a vertical inclination of 0–25 degrees (66% of cavities), with 88% of cavities within a vertical orientation of 0–45 degrees. Only 35 cavities (12%) occurred in trunks or branches with a vertical orientation greater than 50 degrees, and the majority of these occurred at less than 8 m height (median = 4.9 m height, 25–75% interquartile range: 2.6–7.7 m).

Occupancy of cavities was extremely low, although many bird species may not have been nesting at the time that we conducted the cavity surveys. Four of the 289 cavities were found to contain water in the base, and may not have been suitable for use by animals. Of the remaining cavities, only 31 (11%) appeared to be occupied. The most frequent occupants detected in cavities were arthropods (58% of occupied cavities), followed by reptiles and amphibians (19%), birds (16%), and mammals (9%). Of these, the most common occupants were ants, occurring in 39% of the occupied cavities, followed by bees and tree-frogs (10% of occupied cavities each). Only four cavities were found to be occupied by birds during the surveys, with two cavities occupied by the Lilac-crowned Parrot (*Amazona finschi*), and one cavity each occupied by the Mottled Owl (*Ciccaba virgate*), Ferruginous Pygmy Owl (*Glaucidium brasilianum*), and the Collared Forest Falcon (*Micrastur semitorquatus*). Spiders and snakes (*Boa constrictor* and *Oxybelis aeneus*) occupied two cavities each, and the other 7 occupants were detected in only one cavity each.

### Cavities suitable for birds

We found a total of 154 cavities potentially suitable for birds, though if we eliminate 14 cavities found to contain water or occupied by bees and ants, we obtained a total of 140 cavities suitable for birds, giving a density of 37 suitable cavities/ha. Hence, at least 52% of the cavities available in tropical dry forest were unsuitable for cavity-nesting birds. It should be noted however that a number of cavity openings were already excluded from the data as they had a depth <8 cm, and in many cases were found to consist of only a cavity opening with no vertical depth. Therefore, the number and percent of potential cavities observed from the ground that are actually suitable for use by birds is likely to be even lower.

**Figure 2:**
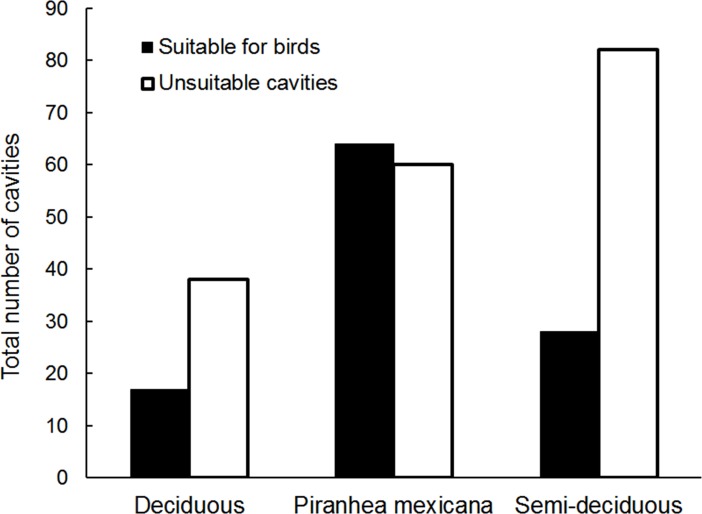
Habitat variation in abundance of cavities suitable for birds. Total number of cavities potentially suitable or unsuitable for use as nest-sites by secondary cavity-nesting birds in three vegetation types of tropical dry forest.

The greatest proportion of unsuitable cavities occurred in semi-deciduous forest with 74.5% of all available cavities unsuitable for use by birds, followed by 69% of unsuitable cavities in deciduous forest, and 48.4% of unsuitable cavities in *Piranhea* forest. Hence, suitability of cavities was significantly associated with forest type (χ^2^_2_ = 18, p < 0.001), with a higher than expected frequency of suitable cavities in *Piranhea* forest, and lower than expected frequency in semi-deciduous forest (Fig. 2).

The density of suitable cavities for birds varied significantly among vegetation types (Table 1), with the highest density of 64.8 suitable cavities/ha in *Piranhea* forest, followed by 40 suitable cavities/ha in semi-deciduous forest, both of which differed significantly from the 18.4 suitable cavities/ha in deciduous forest (*Piranhea*: q = 13.2, p < 0.001; semi-deciduous: q = 8, p = 0.026). Characteristics of suitable cavities also varied significantly among vegetation types (Table 2), occurring in smaller trees, at a lower height, and with a narrower entrance diameter in deciduous forest compared to semi-deciduous and *Piranhea* forest (Table 2). The majority of cavities suitable for birds were formed naturally, with woodpeckers excavating only 13.5% of suitable cavities, though in semi-deciduous forest 21.7% of suitable cavities were excavated by woodpeckers.

### Standing dead trees

There were a total of 418 standing dead trees, with significant differences among vegetation types in abundance of dead trees (Table 1). The highest density of 207 dead trees/ha occurred in deciduous forest, which differed significantly from the density of 100 dead trees/ha in mono-dominant *Piranhea* forest (q = 31.8, p < 0.001), and 47 dead trees/ha in semi-deciduous forest (q = 40, p < 0.001). However, dead trees in deciduous forest had a significantly smaller diameter, being on average <10 cm dbh (Table 2), compared to dead trees in semi-deciduous forest (q = 28.8, p < 0.001), and *Piranhea* forest (q = 22.7, p < 0.001). On average dead trees reached a height of 4–5 m, and this was lower in deciduous forest but only marginally significant (Table 2).

When we restricted the analysis to snags ≥10 cm dbh and ≥2 m height [9], we found a total of 211 snags, giving 56 snags/ha in tropical dry forest. The density of snags did not differ significantly among vegetation types (Table 2), with 70.4 snags/ha in deciduous forest, 57.6 snags/ha in *Piranhea* forest, and 40.8 snags/ha in semi-deciduous forest.

Snags in tropical dry forest had an overall mean 18.4 ± 8.3 cm dbh and 5.6 ± 2.7 m height. However, the diameter of snags varied significantly among vegetation types, being smaller in deciduous forest (Table 2), compared to snags in semi-deciduous (q = 6.2, p < 0.001), and *Piranhea* forest (q = 4.6, p < 0.001). Snags reached an average height of 5–6 m, which did not differ among vegetation types (Table 2).

**Figure 3:**
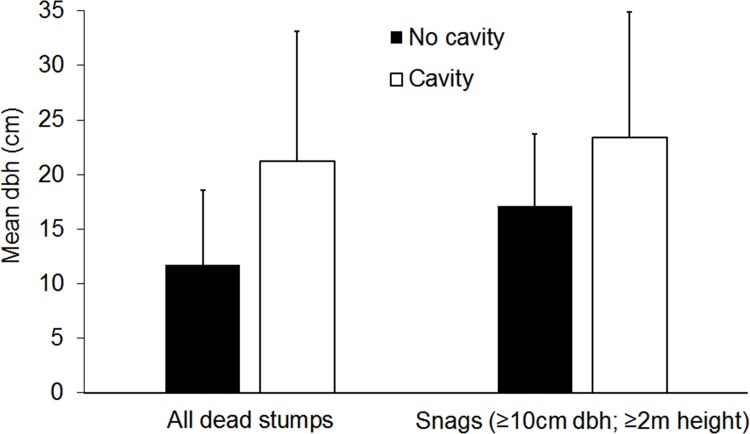
Association of cavities with large standing dead trees. Mean (±SD) diameter at breast height (dbh) of all dead stumps and snags (≥10 cm dbh; ≥2 m height) containing cavities and those without cavities.

Presence of cavities in dead trees was predicted by the multiple logistic regression model of size and height for both all dead stumps, and snags of ≥10 cm dbh; ≥2 m height. In both cases, dbh of standing dead trees significantly predicted the presence of cavities (all stumps: Wald χ^2^_1_ = 27.4, p < 0.001; snags: Wald χ^2^_1_ = 9.9, p = 0.002). Dead trees of a greater size were more likely to contain cavities (Fig. 3), with a 1 cm increase in dbh raising the odds that a dead tree would contain a cavity by 1.105 for all dead stumps and 1.028 for snags. Dead trees with cavities had a mean dbh of 21.2 ± 11.9 cm (interquartile range: 13.3–18.8 cm) for all stumps, or 23.3 ± 11.6 cm (interquartile range: 15.0–26.2) for snags, which was greater than the mean dbh of dead trees in deciduous forest (Table 2). Therefore, while dead trees were more abundant in deciduous forest (Table 1), they were of a significantly smaller size (Table 2; interquartile range: 6–11 cm dbh), and less likely to contain cavities (Figs. 2 and 3).

### Relation with latitude

**Figure 4:**
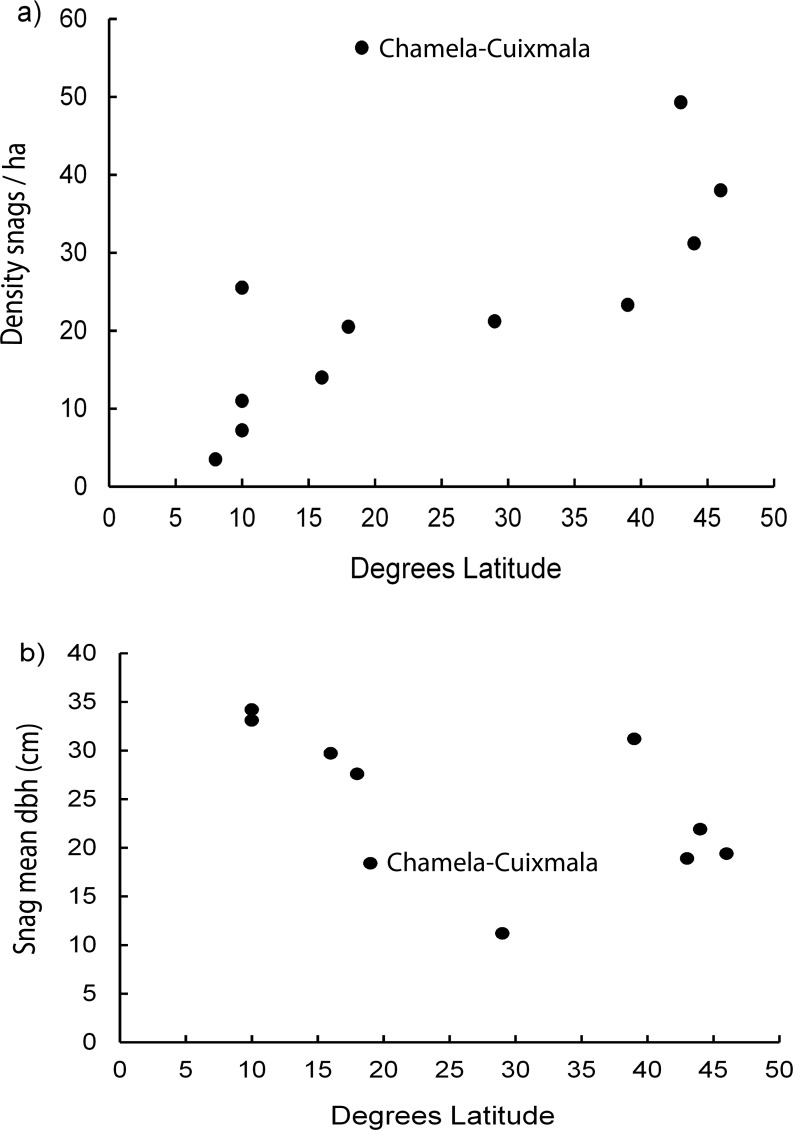
Correlation of snag density and mean dbh with latitude. Location of data from the Chamela-Cuixmala site in the correlation of a) snag (≥10 cm dbh, ≥2 m height) density/ha, and b) snag mean diameter at breast height (dbh) with latitude for 12 temperate and tropical forest sites. Additional data from Gibbs et al. [9].

**Figure 5:**
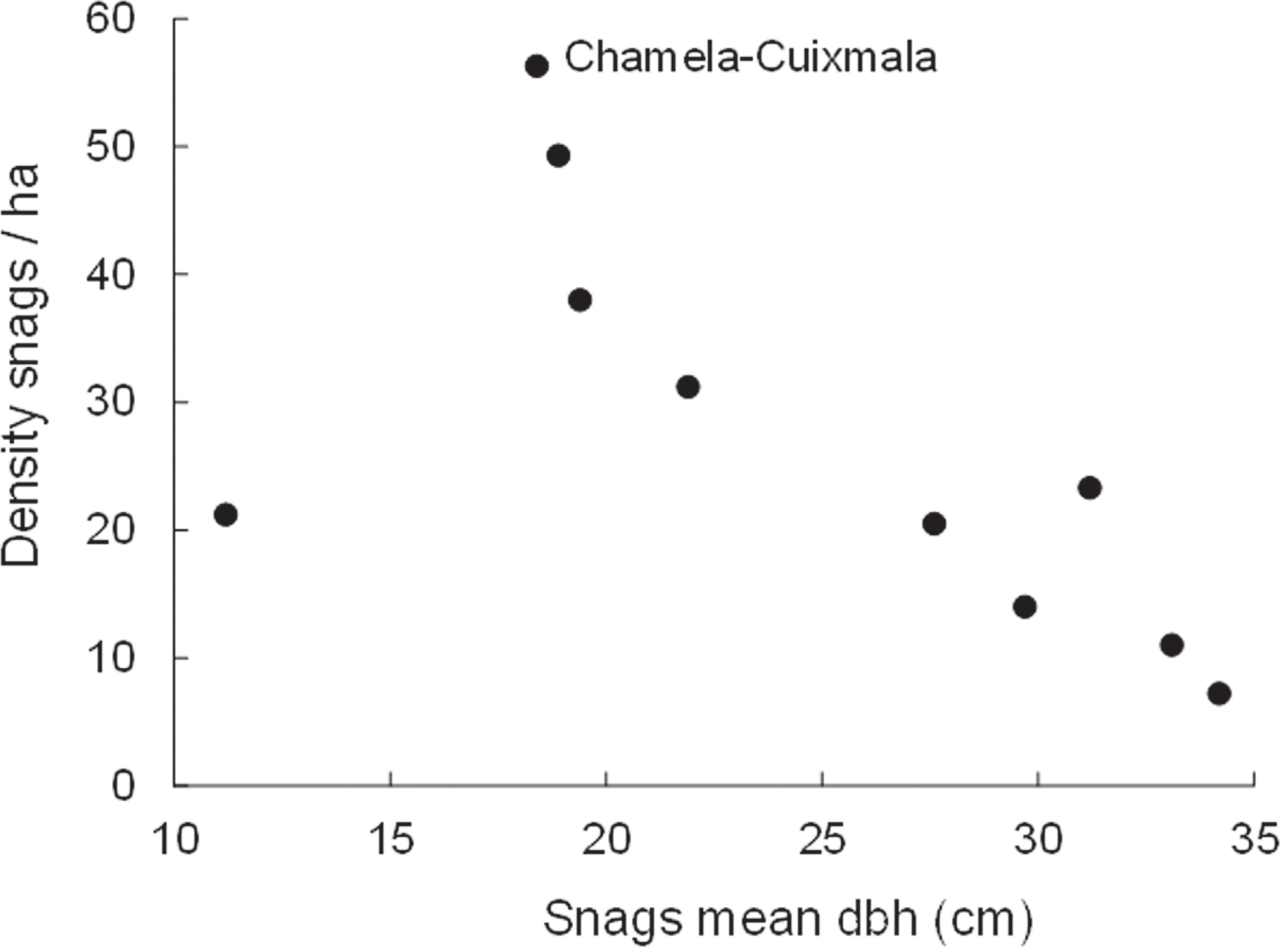
Correlation of snag density with mean dbh at ten forest sites. Density of snags (≥10 cm dbh, ≥2 m height) and mean diameter at breast height (dbh) of dead trees at ten temperate and tropical forest sites, illustrating the data point from the Chamela-Cuixmala site. Additional data from Gibbs et al. [9].

Combining our data to that obtained by Gibbs et al. [9] and Boyle et al. [10] on snag density and mean dbh in 12 temperate and tropical forests, found that the significant correlation of snag density with latitude was maintained (r = 0.617, df = 11, p = 0.043), though the Chamela-Cuixmala site had the highest density of 56.3 snags/ha recorded for any tropical or temperate forest (Fig. 4A). By comparison, the correlation of snag mean dbh with latitude was no longer significant (r = 0.52, df = 10, p = 0.12), and while the Chamela-Cuixmala site had a low mean 18.3 cm snag dbh, this was intermediate between sites in Belize and Florida (Fig. 4B; S1 Table). Moreover, we determined that snag density was significantly correlated with mean snag dbh (r = 0.65, df = 10, p = 0.04), where forest sites with a lower density of snags generally had snags of larger dbh (Fig. 5). However, the multiple regression model found that neither latitude nor mean dbh significantly predicted snag density in the ten forest sites (Model r^2^ = 0.48, F_2,7_ = 3.3, p = 0.10).

## Discussion

### Cavity availability and characteristics

Tropical dry forest had a high density of 77 cavities/ha, though over half of these were not suitable for use by birds, giving a density of 37 cavities suitable for birds/ha. Tree cavities were also relatively more abundant in small dispersed patches of mono-dominant *Piranhea mexicana* forest, and semi-deciduous forest. Even if we consider only the dominant vegetation of deciduous forest, then there was still a high density of 44 cavities/ha, or 18.4 bird suitable cavities/ha. Recent studies suggest a negative correlation of cavity density with latitude [10]; presenting high densities of cavities in tropical forests due to environmental conditions of higher temperatures and humidity which could accelerate natural decay processes [7]. The cavity density recorded in our study follows this pattern of high cavity density in tropical forests, although our estimate of cavity density in tropical dry forest was much higher than that reported for similar studies in other tropical forests, such as tropical moist forest in Argentina with 16.8 cavities/ha [25] and 4.5 suitable cavities/ha [2], or the 13.6 cavities/ha (4.1 suitable cavities/ha) in subtropical piedmont forest, and 12.8 cavities/ha (3.9 suitable cavities/ha) in cloud forest, both in the Andes of Argentina [31].

By comparison, very high densities of 407 cavities/ha were recorded in old-growth humid semi-deciduous tropical forest in Thailand [32], and 111.7 cavities/ha in tropical rainforest of Costa Rica [10]. However, neither of these two studies considered cavity depth in the criteria for cavity definition. In fact, very few studies of tree-cavity density have measured cavity depth, and this was considered as a criterion by only five studies included in the analysis by Boyle et al. [10]. Of studies conducted in tropical forests, only the study in tropical moist forest of Argentina has incorporated cavity depth as a criterion [2]. Cavity depth is one of main selection criteria for use of cavities by birds [3]. Furthermore, as noted by Cockle et al. [3] and observed in the present study, a high percent of cavity openings observed from the ground were less than 8 cm deep, making them unsuitable for use by birds and many other animals. Surveys of cavity abundance from the ground which do not determine depth of cavities may be highly misleading as to the suitability of cavities for use by secondary cavity-nesters [3]. Ground surveys tend to over-estimate cavity abundance [3, 33], particularly for large trees [34], and may under-estimate cavity abundance in trees of smaller girth [34].

We also found that cavities suitable for use by birds were not homogenously distributed among the vegetation types of tropical dry forest, being concentrated at highest densities in mono-dominant *Piranhea mexicana* forest. However, deciduous forest is the dominant vegetation type, comprising 86% of tropical forest vegetation in the region [18], while semi-deciduous and mono-dominant *Piranhea* forests occur in small discontinuous patches within the deciduous forest mosaic [21, 22]. Furthermore, cavities suitable for birds in deciduous forest occurred in smaller trees, at a lower height, with a smaller entrance diameter, suggesting they may only be suitable for smaller-sized birds, and would not provide nesting resources for larger secondary cavity nesters which require large, high cavities [35, 11].

As found in other tropical forests [12, 7], the great majority of cavities in tropical dry forest were formed naturally, though with only 8.6% of all cavities excavated by woodpeckers at the Chamela-Cuixmala tropical dry forest site, this was much lower than the 20–30% of cavities excavated by woodpeckers at two other tropical forest sites [7]. Hence, in contrast with temperate forests, woodpeckers do not appear to be important generators of cavity formation in tropical dry forest. Nevertheless, woodpeckers excavated a slightly higher 13.5% of cavities suitable for birds, and a greater proportion of cavities in semi-deciduous forest were excavated by woodpeckers. Hence, woodpeckers may play a greater role in the formation of cavities for secondary cavity-nesters within specific vegetation types of tropical dry forest. Furthermore, woodpeckers may play a role in natural cavity formation by breaking open bark and introducing fungal or disease pathogens during foraging activities. The number of cavities created by woodpeckers may also be underestimated given that woodpeckers tend to excavate cavities in dead trees or branches [36, 3], which would be lost more rapidly than cavities in live trees.

We found a low occupancy of cavities; however we could only determine use of cavities if the occupant was observed at the cavity entrance, therefore we may not have detected all cavity occupants. It may also be that many cavity-nesters were not nesting during the weeks we conducted our surveys. Nevertheless, given the high density of cavities in tropical dry forest there may be ample nesting options for smaller secondary cavity-nesting birds. Wiebe [37] also suggests that cavities are not limiting for birds in mature unmanaged forests. However, secondary cavity-nesters demonstrate species-specific requirements for nest-sites [38, 39], and may be highly selective in their use of cavities [40, 3]. Therefore, it may be that cavity limitation occurs at a species-specific level, particularly for large-bodied secondary cavity-nesters that require larger tree cavities.

The significant difference in cavity characteristics among vegetation types within tropical dry forest means that nesting resources for large avian secondary cavity-nesters, such as parrots, owls and falcons, are concentrated in small, discontinuous patches of semi-deciduous or mono-dominant *Piranhea* forest. Within tropical dry forest, the deciduous and semi-deciduous vegetation types have similar tree species diversity, but with distinct characteristics of species composition, and forest structure, in which large tree species occur mainly in semi-deciduous vegetation [24]. Semi-deciduous forest also provides essential food resources during the dry season [41], highlighting the importance of this habitat type in providing nest-sites and food resources for secondary cavity-nesters during a critical time of the year. However, tropical dry forest in Mexico is suffering high rates of deforestation and disturbance [42, 19], and many of the large, tree species characteristic of semi-deciduous and *Piranhea* forests are logged for construction and the wood-furniture industry [43]. Hence, given that semi-deciduous forest comprises only 14% of tropical forest in the region [18], it is essential to maintain the mosaic of small patches of semi-deciduous and *Piranhea* forests within the larger expanse of deciduous forest vegetation, in order to provide key nesting resources for a diverse community of secondary cavity-nesters.

### Density of snags

The tropical dry forest of the Chamela-Cuixmala site had the highest density of snags recorded for any tropical or temperate forest [9, 10]. Hence, although the positive relation of snag density with latitude from the equator was maintained, the Chamela-Cuixmala site did not follow the pattern of a low density of snags demonstrated by tropical moist or humid forests [9]. Furthermore, while snag density was related to mean dbh of snags, neither latitude nor dbh reliably predicted snag density in ten temperate and tropical forest sites. Therefore, it may be that other environmental factors, such as seasonal patterns of rainfall, temperature, or soil humidity are more likely predictors of snag density.

The Chamela-Cuixmala tropical dry forest experiences low annual rainfall and a severe dry season of 6–8 months [20], which may promote tree death particularly following extremely dry years associated with the El Niño Southern Oscillation in the Pacific Ocean. This could have a more severe impact on trees in deciduous forest vegetation on the hills and slopes, which has sandy loam soils with a low capacity for water retention [44]. Low rainfall with long drought periods, poor soils, and high solar radiation, particularly on dry forest hills [45], means that trees in deciduous forest may be more likely to experience high water stress during the prolonged drought of the dry season [44]. Deciduous forest in Chamela-Cuixmala has an annual mortality rate of 55 trees/ha [44], resulting in the significantly higher density of 70 snags/ha in deciduous forest vegetation.

However, although there was a higher density of snags in deciduous forest vegetation, these had significantly smaller diameter, and snags of a larger diameter were more likely to contain cavities. It may be that smaller snags are not of the size preferred by primary cavity excavators [36], therefore, although there may be a greater density of snags in deciduous forest there may be few large-sized snags suitable for use as a nesting substrate by primary cavity excavators.

### Latitudinal relationships in cavity and snag densities

Tropical dry forest did not follow the pattern of low snag density in tropical forests [9], but did fit the trend of a high density of naturally-formed cavities in forests closer to the equator [10]. It is unclear therefore whether these relationships with latitude are accurate or an artifact of the concentration of studies in moist or wet tropical forests. As found in the present study, tropical dry forest does not necessarily follow the patterns exhibited by moist and wet tropical forests. Hence, as with floristic diversity within Neotropical forests which is related to precipitation [46], other environmental factors may drive snag and cavity densities in tropical and temperate forests. There are a diversity of forest types within the tropics, therefore we need more studies of cavity and snag availability within tropical forests in order to evaluate the factors driving availability of these specific nesting resources.

The combination of environmental factors at the Chamela-Cuixmala site may create unique conditions resulting in higher than expected snag and cavity densities. Floristic diversity in the Chamela-Cuixmala tropical dry forest is also higher than that recorded for any other dry forest site [47], and is much greater than that predicted for its low precipitation [24]. This low annual precipitation with long dry periods results in high water stress for trees in tropical dry forest [44], with increased risk of branch or tree death, resulting in breakages where cavities may form, or a high number of standing dead trees. The small diameter of many trees in dry forest [24] also means these may be less likely to fall and may remain standing.

The Pacific coast of Mexico is subject to a high frequency of hurricanes, and the number making landfall has increased over the last few decades, mainly level 1 or 2 hurricanes [48]. It is unclear how this may influence snag and cavity densities in Pacific coast forests, though studies in Australian forests indicate that damage caused by cyclones can increase cavity availability [49]. Hence, in the short-term, the impact of hurricanes may reduce snag and cavity densities as a result of tree-fall. However, the damage caused to broken trunks and branches may in the long-term facilitate the formation of cavities. Therefore, the high density of snags and natural cavities in the Chamela-Cuixmala tropical dry forest may be a reflection of its climatic and physical conditions, and a past history of low level hurricane impacts.

### Implications for cavity nesting birds

We found a high density of snags and cavities in tropical dry forest, but these demonstrate a pattern of high spatial aggregation, being closely associated with semi-deciduous and mono-dominant *Piranhea mexicana* forests. In particular, cavities and snags suitable for use as nesting resources by birds are concentrated in these small discontinuous patches of vegetation within the deciduous forest mosaic. This may restrict the availability of these nesting resources, as territorial behavior of nesting pairs would limit access to nest-sites where these are clumped in distribution [50, 51, 52].

Given the high density of snags and cavities in the Chamela-Cuixmala tropical dry forest we may also expect a high number of avian cavity-nesters. While the Chamela-Cuixmala site has a higher species richness of cavity-nesting birds compared to temperate forests, this is lower than that for tropical moist and wet forests [17], and the proportion of species that are cavity-nesters, or of cavity-nesters that are primary excavators, is lower than that for other tropical or temperate forests [9]. However, in order to evaluate the relation of snag and cavity density with the community of cavity-nesters it is important to take into consideration density of cavity-nesters, not just species richness. The high spatial aggregation of snag and cavity resources in the heterogeneous tropical dry forest of Chamela-Cuixmala, and the specific requirements of many cavity-nesters, may also mean that actual resource availability for cavity-nesters is lower than may appear.

Our results highlight the importance of maintaining the different vegetation types within the tropical dry forest mosaic to provide nesting resources for a variety of primary and secondary cavity-nesters. Small patches of semi-deciduous and mono-dominant *Piranhea* forests are particularly at risk due to selective logging and clearance for agriculture or pasture-lands. This is reflected in rates of forest loss, where semi-deciduous forest has almost twice the deforestation rate of deciduous forest along the coast of Jalisco [53]. As determined by the characteristics of snags and cavities, there is a concentration of resources suitable for use by birds in these discontinuous forest patches. In particular, large-bodied secondary cavity-nesters may be limited by the spatial aggregation of a reduced number of cavities suitable for nesting.

## Supporting Information

S1 Table Description of sites used for comparison of snag density and dbh, obtained from Gibbs et al. [9]. (DOCX)
