# Supplementary material for: High Density of Tree-Cavities and Snags in Tropical Dry Forest of Western Mexico Raises Questions for a Latitudinal Gradient
Source: PLoS One. 2015 Jan 23;10(1):e0116745. doi: 10.1371/journal.pone.0116745 (PMC4304802; doi:10.1371/journal.pone.0116745)
Supplement: S1 Table — (DOCX) [file pone.0116745.s001.docx]

Table S1: Description of sites used for comparison of snag density and dbh, obtained from Gibbs et al. (1993).

| Site | State, Country | Latitude | Snag density / ha | Mean dbh (cm) | Mean annual rainfall (mm) | Forest type |
| --- | --- | --- | --- | --- | --- | --- |
| Caño Caracol | Guárico, Venezuela | 7^0^ 56’ | 3.5 | ND | 1,350 | Gallery forest in tropical wet savannah |
| Monteverde Cloud Forest Reserve | Puntarenas-Alajuela, Costa Rica | 10^0^ 18’ | 7.2 | 34.2 | 2,579 | Low montane wet forest |
| La Selva Biological Station | Heredia, Costa Rica | 10^0^ 25’ | 11 | 33.1 | 4,351.6 | Lowland tropical wet forest |
| Bladen Branch Nature Reserve | Toledo, Belize | 16^0^ 23’ | 14 | 29.7 | 4,064 | Subtropical wet forest |
| Rio Bravo Conservation and Management Area | Orange Walk, Belize | 18^0^ 02’ | 20.5 | 27.6 | 1,574.5 | Subtropical moist broadleaf forest, |
| Chamela-Cuixmala Biosphere Reserve | Coastal Jalisco, Mexico | 19^0^ 35’ | 56.3 | 18.4 | 780 | Tropical dry forest |
| Ocala National Forest | Florida, USA | 29^0^ 10’ | 21.2 | 11.2 | 1,400 | Long-leaf Pine forest |
| Glover-Archibold Park | Washington DC, USA | 38^0^ 55’ | 23.3 | 31.2 | 981.1 | Northeastern coastal-plain mixed hardwood forest |
| Holt Research Forest | Arrowsic, Maine, USA | 43^0^ 52’ | 49.3 | 18.9 | 1,140 | Pine-oak forest |
| White Mountain National Forest | New Hampshire, USA | 44^0^ 06’ | 31.2 | 21.9 | 1,750 | Northern hardwood forest |
| Baxter State Park | Northern Maine, USA | 45^0^ 59’ | 38 | 19.4 | 939.8 | Spruce-fir and northern hardwood forests |
